# Iodide Protects Heart Tissue from Reperfusion Injury

**DOI:** 10.1371/journal.pone.0112458

**Published:** 2014-11-07

**Authors:** Akiko Iwata, Michael L. Morrison, Mark B. Roth

**Affiliations:** Fred Hutchinson Cancer Research Center, Division of Basic Sciences, Seattle, Washington, United States of America; Emory University, United States of America

## Abstract

Iodine is an elemental nutrient that is essential for mammals. Here we provide evidence for an acute therapeutic role for iodine in ischemia reperfusion injury. Infusion of the reduced form, iodide, but not the oxidized form iodate, reduces heart damage by as much as 75% when delivered intravenously following temporary loss of blood flow but prior to reperfusion of the heart in a mouse model of acute myocardial infarction. Normal thyroid function may be required because loss of thyroid activity abrogates the iodide benefit. Given the high degree of protection and the high degree of safety, iodide should be explored further as a therapy for reperfusion injury.

## Introduction

Metabolic rate in animals or rate of oxygen consumption varies widely depending on conditions [1]. Changes in oxygen consumption create a corresponding need for reducing activity to maintain the appropriate chemical environment required to sustain life [2]. This balance between oxidizing and reducing potentials is often lost in pathological states caused by injury and disease. For example, when heart tissue is temporarily deprived of oxygen during a heart attack, the rate of oxygen consumption decreases, when blood flow is restored, oxygen consumption increases to a degree several fold higher than prior to the ischemic event [3]. During this period of excessive oxygen consumption post reperfusion, considerable damage can be done to the heart [4]. Many results suggest that damage to the heart is caused by altered redox chemistry that, in turn, instigates a process of inflammation and cell death [5]. This view of the vulnerability of the heart during reperfusion led researchers at the NIH New Horizons in Cardioprotection workshop to state that a primary goal of heart medicine should be to prevent the heart from “metabolizing itself to death” [6].

Previously we showed that elemental reducing agents such as sulfide and selenide can decrease metabolism and improve outcome in preclinical models of oxygen deprivation and ischemia reperfusion injury [7], [8]. Iodide is also an elemental reducing agent. When added to a solution of hydrogen peroxide, iodide catalytically converts hydrogen peroxide to water and oxygen [8], [9]; when added to plasma, iodide increases peroxidase activity [10]. These facts led us to test whether iodide could be used as a therapy to preserve and protect heart tissue from damage caused by ischemia reperfusion injury.

## Methods

All experiments in this study were designed and performed in accordance with federal guidelines (Guide for the Care and Use of Laboratory Animals, (2011) National Research Council, National Academies Press, Washington D.C.) and approved by the Institutional Animal Care and Use Committee at Fred Hutchinson Cancer Research Center (OLAW assurance number A3226-01). All experiments were conducted at the Fred Hutchinson Cancer Research Center (Seattle, WA). During and after surgery, anesthesia and analgesia were administered to alleviate pain and suffering (see below in myocardial ischemia reperfusion section).

### Study Design and Statistics

The experiments described in this study were exploratory. Therefore, sample sizes were variable and based on variability of outcome. Because all data was collected from live animals or discrete time endpoints, only animals that died prematurely were excluded from analysis. Statistical analyses were performed using GraphPad Prism or Microsoft Excel software. Differences between groups were evaluated using one-way ANOVA, followed by post hoc Tukey test, or two-tailed Student's t-test. P values <0.05 were considered statistically significant. For the blinded myocardial ischemia reperfusion experiments, vials containing solutions of either sodium iodide or control saline were made by one person, encoded by another person, and the experiments carried out be a third person. Once the result was complete and seen by all parties the code was revealed.

### Myocardial Ischemia Reperfusion (MIR)

**Figure 1:**
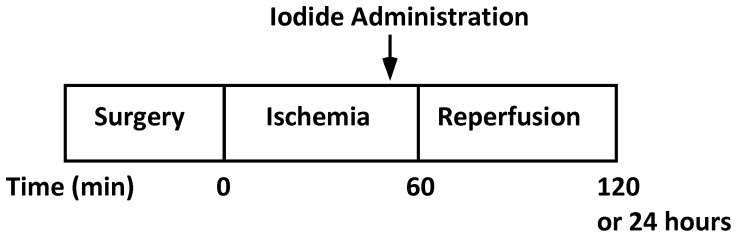
Acute myocardial infarction model timeline. Mice were subjected to 60 minutes of ischemia followed by 120 minutes or 24 hours of reperfusion. Animals were infused with test agents or saline (sodium chloride) 5 minutes before the end of the ischemic period and before reperfusion.

The timeline for the MIR procedure is presented in Figure 1. Mice were anesthetized with ketamine/xylazine mixture (100 mg/Kg, and 10 mg/Kg body weight, respectively) intraperitoneal injection, their tracheae intubated, and placed on mechanical ventilation set at a tidal volume 220 µL and a rate of 100 breaths per minute using 2% isoflurane in 100% oxygen. A left thoracotomy was performed; the left anterior descending (LAD) artery located and ligated with the use of a 7-0 silk suture at approximately 2–3 mm from the tip of the left auricle. A small piece of polyethylene tubing (PE-10) was used to secure the ligature taking care to prevent damage to the artery. Coronary occlusion and reperfusion were confirmed by visual inspection under a dissecting microscope by observing color changes of the tissue. Mice were subjected 60 min of myocardial ischemia followed by 120 minutes of reperfusion. After reperfusion, mice were euthanized by exsanguination under anesthesia. The LAD was again ligated at original location and 1.5% Evans blue dye (Sigma) was perfused before the heart was harvested. The infarct size was evaluated by double staining of Evans blue dye and 1% triphenyltetrazolium chloride (TTC, Sigma). Sterile surgical technique was used for animals that survived for 24 hours or more and they received buprenorphine every 8 hours as need for analgesia.

### Troponin I Measurement

A blood sample (500 µL) was collected from mice prior to the Evans blue dye perfusion through a catheter placed in the carotid artery. Plasma was separated by centrifugation and the levels of the cardiac-specific isoform of Troponin-I (ng/mL) were assessed using an ELISA kit from Life Diagnostics (West Chester, PA).

### Neutrophil Counts

Heart sections were fixed in 10% formalin, embedded in paraffin blocks, and cut into 2 µm sections. Sections were stained with hematoxylin/eosin and examined by light microscopy (×600). The number of neutrophils in the myocardium in each field was counted and normalized to the area of the field. Neutrophil counts were made on three sections per mouse heart and on five fields per section.

### Echocardiograhic Assessment

In vivo transthoracic echocardiography of the left ventricle (LV) was performed using a 30-MHz RMV scanhead interfaced with a Vevo 770 (Visualsonics) and two-dimensional images were obtained. Mice were anesthetized with 1–3% isoflurane. Heart rate was maintained between 350 and 450 bpm and body core temperature was maintained at 36–38°C by adjusting isoflurane concentration and ambient heat using a heat lamp. End-diastolic diameter (EDD) and LV end-systolic diameter (ESD) were measured the day before the myocardial I/R procedure for a baseline and one and 4 weeks after the procedure. From these data LV percent fractional shortening (FS) and LV ejection fraction (EF) were then calculated.

### Oral Administration of Sodium Iodide

Water containing either 0.028 or 0.28 mM sodium iodide was used to replace the water of animals 2 days before the MIR procedure. By measuring consumption we found that the iodide containing water was consumed at the same rate as normal water, resulting in an increase in iodide consumption of 10 or 100 times the normal diet.

### Measurement of Heart Rate and Body Core Temperature

Heart rate (HR) and body core temperature (Temp) were measured non-invasively, in vivo and in real-time using radio telemetry. Physiotel HD-X11 transmitters (Data Sciences International) were surgically implanted into mice according to manufacturer's protocol. Measurements of HR and Temp after iodide administration were performed 4–6 weeks after transmitter implantation surgery.

### Measurement of Carbon Dioxide Production

Mice were placed in a closed 0.5 L chamber supplied with 0.5 L/min room air from which CO_2_ was removed. Outflow gas was passed through a Licor Li7000 to measure CO_2_.

### Reduction of Thyroid Activity

Mice were fed iodide deficient chow supplemented with 0.15% propylthiouracil (PTU) from Harlan-Teklad (TD.95125) for three months before being used for MIR procedure.

## Results

**Figure 2:**
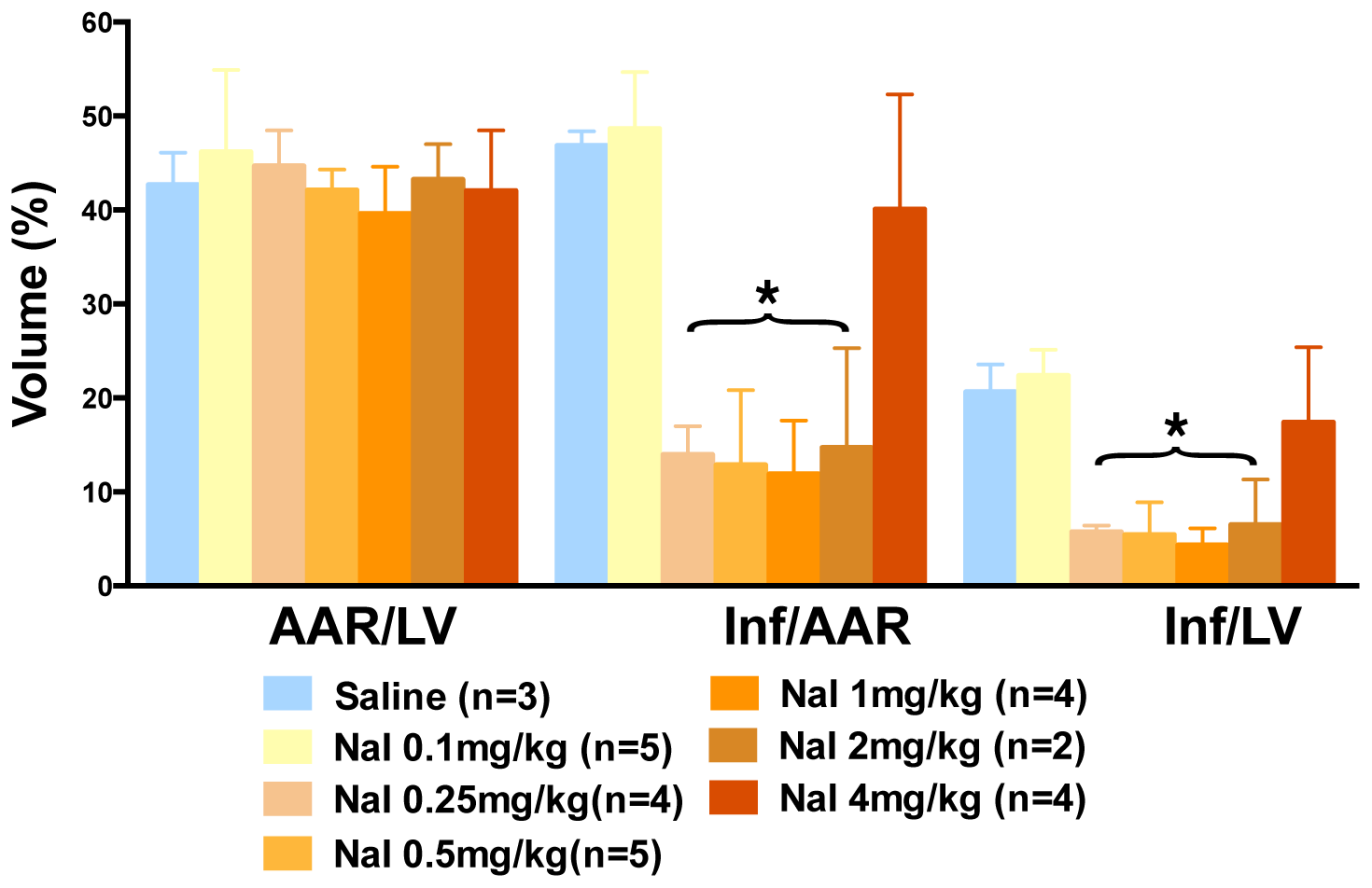
Iodide reduces heart damage after MIR. Infarct (Inf) and area at risk (AAR) volumes were measured using morphometry after administration of escalating doses of iodide. Y-axis is volume as a percent of total. X-axis labels are AAR/LV (ratio of area at risk volume to left ventricle volume), Inf/AAR (ratio of infarct volume to area at risk volume) and Inf/LV (infarct volume to left ventricular volume). Colored bars describe groups that are listed in the key. The blue bars are saline. All other bars describe groups of animals that received iodide at different concentrations in mg/kg. Parenthetical n numbers define group sizes. The two asterisk brackets over Inf/AAR and Inf/LV are meant to show that all 4 groups under the brackets showed statistically significant differences when compared to control saline (p<0.05).

**Figure 3:**
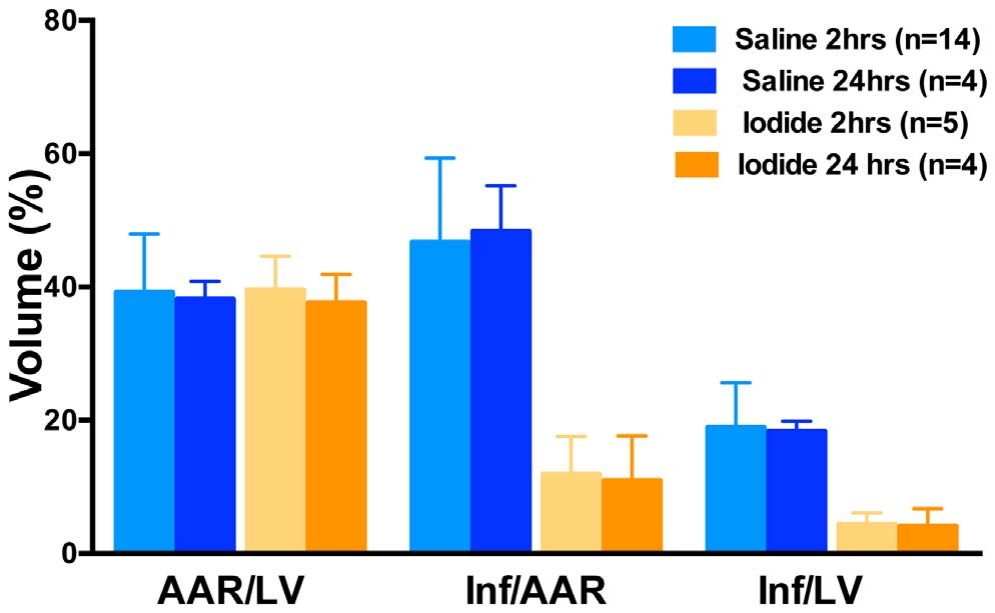
Iodide reduces heart damage after both 2 and 24 hours of reperfusion. Infarct (Inf) and area at risk (AAR) volumes were measured using morphometry after administration of iodide. Y-axis is volume as a percent of total. X-axis labels are AAR/LV (ratio of area at risk volume to left ventricle volume), Inf/AAR (ratio of infarct volume to area at risk volume) and Inf/LV (infarct volume to left ventricular volume). The data from 2 hours is the same as shown in Figure 2. The asterisks indicate a statistically significant difference between saline and iodide treated animals (p<0.05).

**Figure 4:**
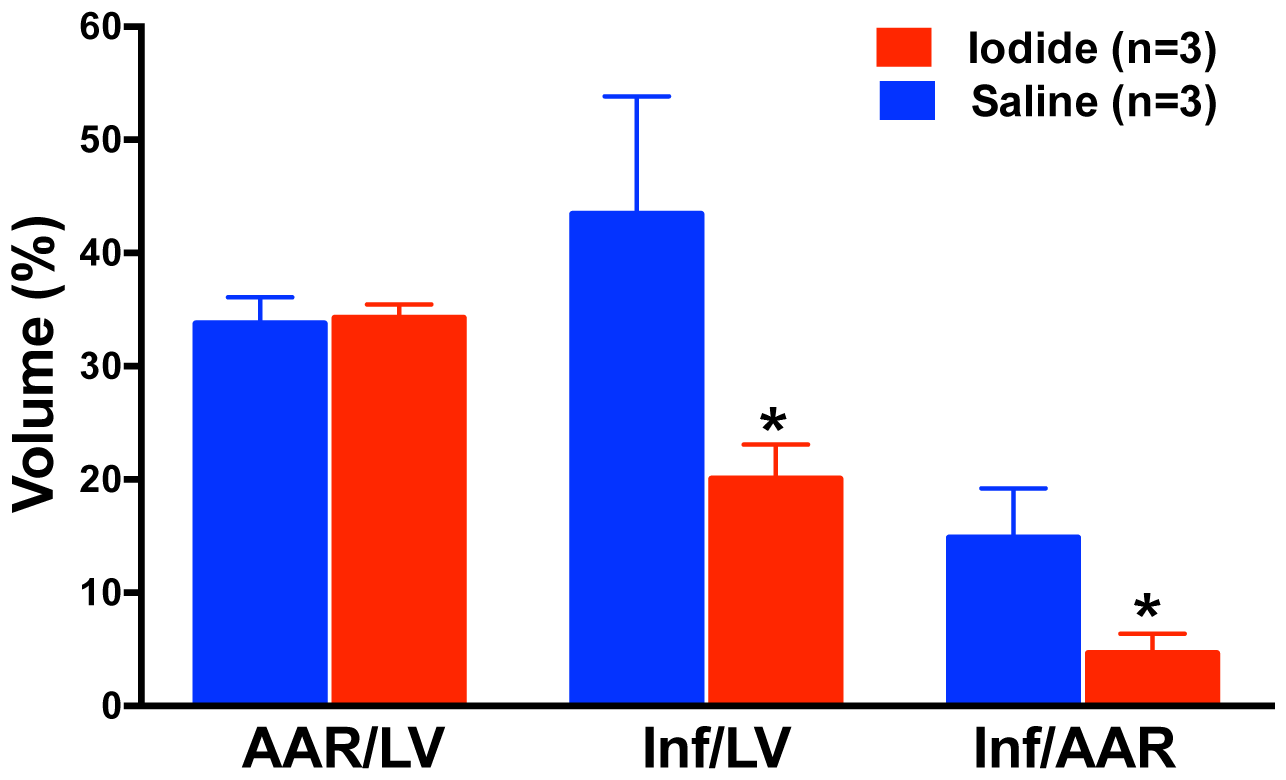
Measurement of heart damage after blinded iodide treatment. Infarct (Inf) and area at risk (AAR) volumes were measured using morphometry after administration of 1 mg/kg iodide. Y-axis is volume as a percent of total. X-axis labels are AAR/LV (ratio of area at risk volume to left ventricle volume), Inf/AAR (ratio of infarct volume to area at risk volume) and Inf/LV (infarct volume to left ventricular volume). Blue bars describe the data from control animals. Red bars describe the data from iodide treated animals. Parenthetical n numbers define group sizes. Inf/LV and Inf/AAR showed statistically significant differences between saline and iodide treated animals (p<0.05).

To first test the effect of sodium iodide (referred to here as iodide or NaI) in acute myocardial infarction we used a myocardial ischemia reperfusion (MIR) model described in the methods and presented as a timeline in Figure 1. At the end of the procedure the size of the infarcted tissue was determined. When animals were infused with iodide (see color coded key) we observed a 75% decrease in the size of the infarction compared to control saline (Figure 2). This degree of statistically significant benefit is evident in four groups of animals (see brackets) with concentrations of iodide delivered ranging over approximately 1 order of magnitude from 250 µg/kg to 2 mg/kg. Similar benefit was seen when reperfusion was allowed to proceed for 24 hours (Figure 3). Since these experiments were randomized but not blinded, we performed a smaller blinded study comparing control saline to 1 mg/kg iodide with 2 hours of reperfusion and observed similar results (Figure 4).

**Figure 5:**
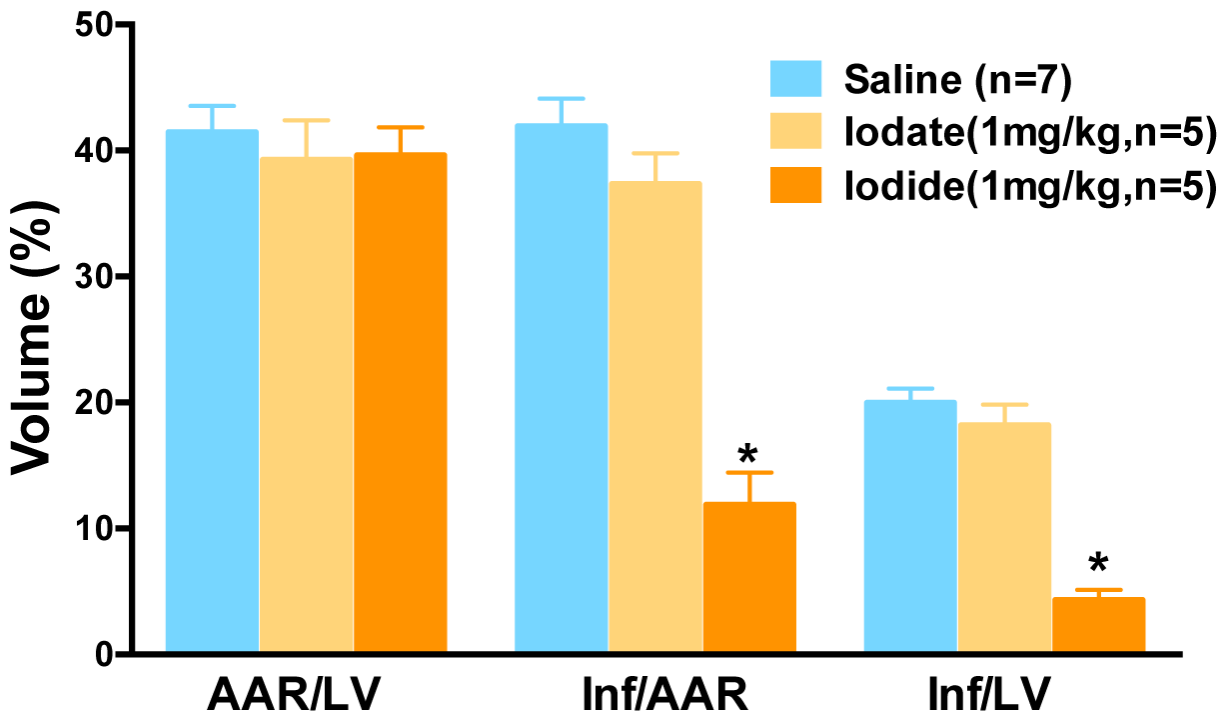
Oxidized iodine, iodate, does not reduce heart damage after MIR. Infarct (Inf) and area at risk (AAR) volumes were measured using morphometry after administration of iodate and iodide. Y-axis is volume as a percent of total. X-axis labels are AAR/LV (ratio of area at risk volume to left ventricle volume), Inf/AAR (ratio of infarct volume to area at risk volume) and Inf/LV (infarct volume to left ventricular volume). Colored bars describe groups that are listed in the key. The blue bars are saline. Animals that received either 1 mg/kg iodate (tan) or iodide (orange). Parenthetical n numbers define group sizes. The two asterisks indicate that iodide shows a statistically significant difference when compared to control saline (p<0.05).

We also tested whether the oxidized form of iodine, iodate in the form of sodium iodate, would provide benefit in the MIR model. We observed that iodate does not provide benefit (Figure 5).

**Figure 6:**
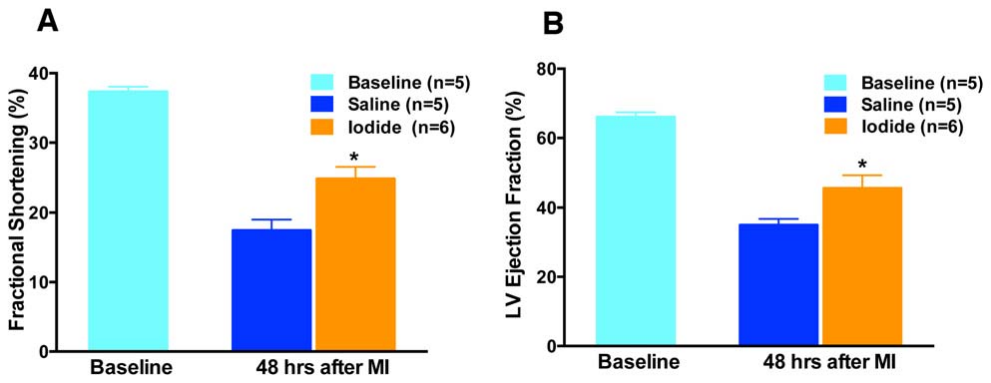
Echocardiography 48 hours post MIR and iodide treatment. Fractional shortening and LV ejection fraction before (baseline) and at 48 hours reperfusion following the MIR procedure with and without iodide (1 mg/kg) administration. Animals were survived following the MIR procedure for 48 hours and then subjected to echocardiography. Fractional shortening (A) and ejection fraction (B) are presented. * P<0.001 vs. saline.

**Figure 7:**
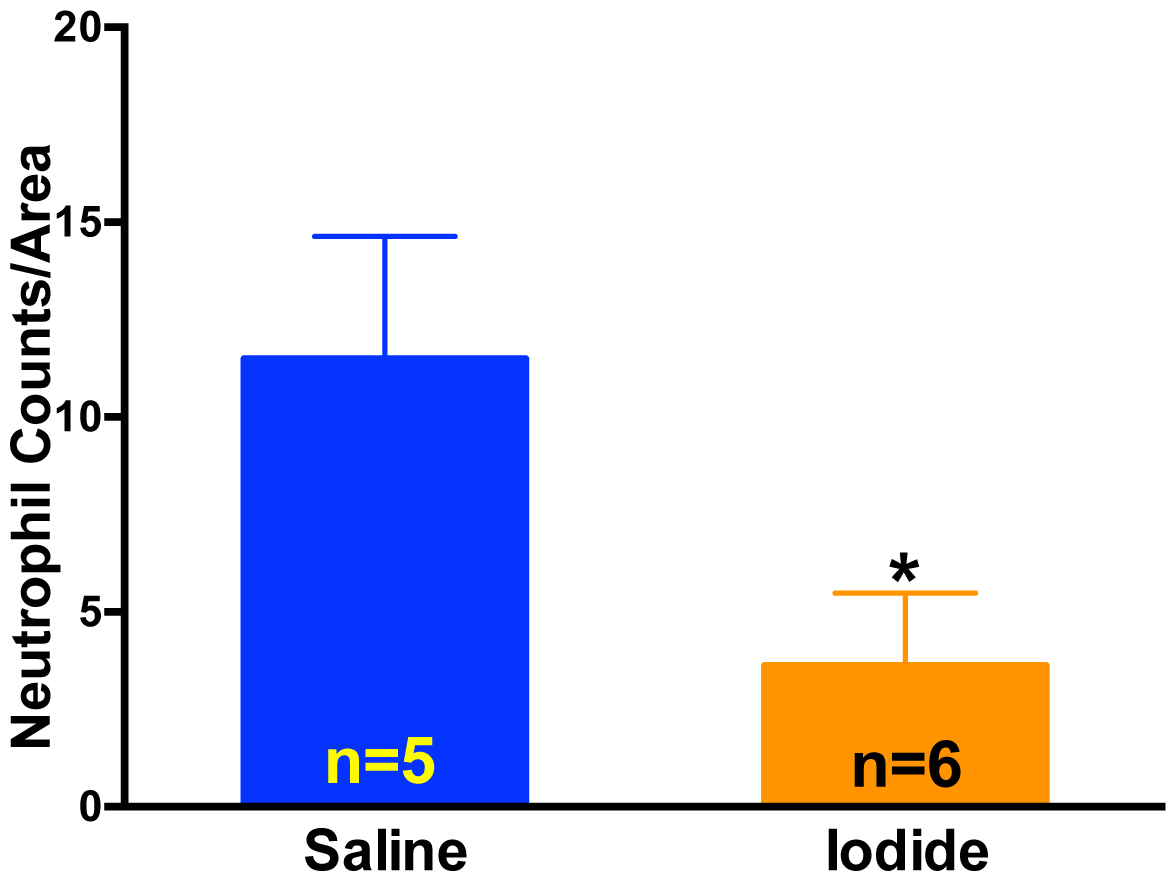
Neutrophil accumulation after MIR and iodide treatment. Mice were subjected to MIR with 2 hours of reperfusion. At 5 min prior to reperfusion, mice were given either iodide (1 mg/kg) or saline by retro orbital injection. Hearts were sectioned following reperfusion and neutrophils quantitated. *p<0.05.

**Figure 8:**
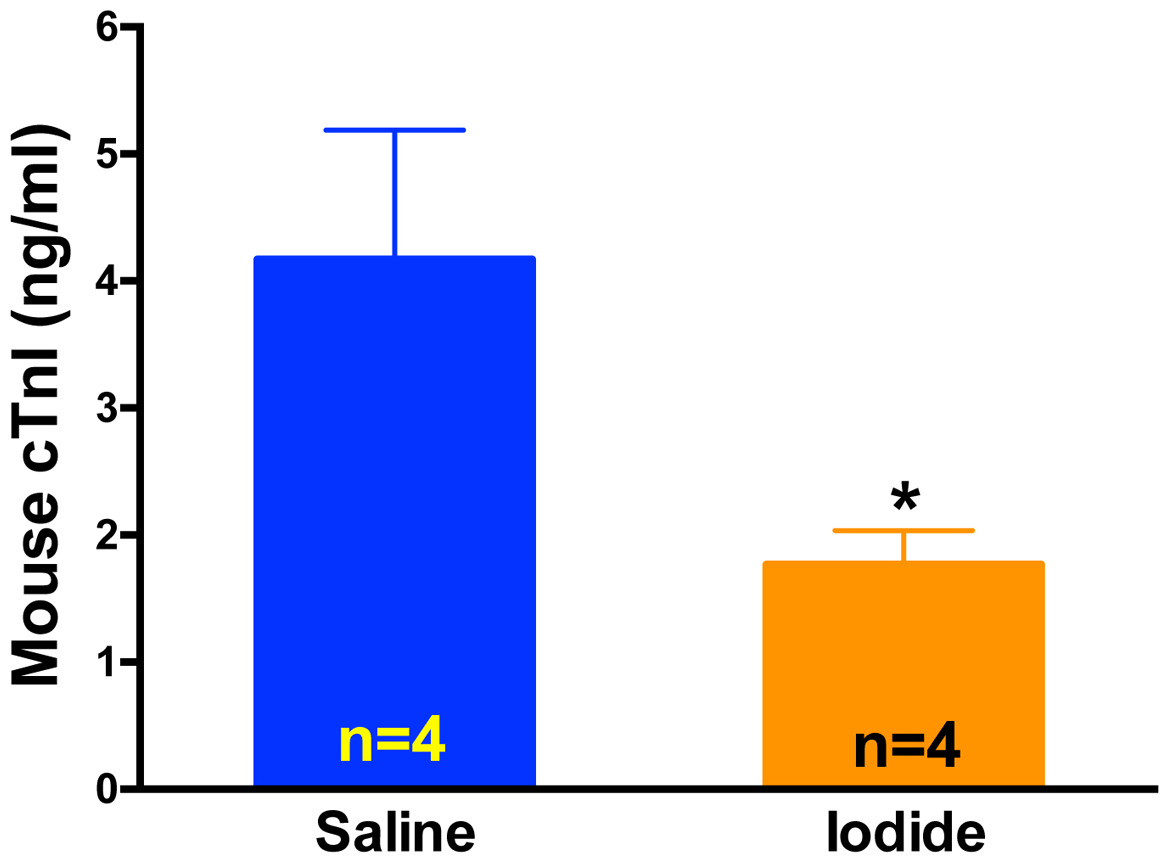
Plasma troponin I measurement after MIR and iodide treatment. Mice were subjected to LAD ligation for 60 min following 2 hours of reperfusion. At 5 min prior to reperfusion, mice were given either iodide (1 mg/kg) or saline by retro orbital injection. Following 2 hours of reperfusion, blood was collected and plasma cardiac troponin-I (cTnI ng/mL) measured by ELISA. * p<0.05.

To further test the hypothesis that iodide protects heart tissue from reperfusion injury we conducted several other assays including echocardiography to test function (Figure 6), neutrophil accumulation in the area at risk to gauge inflammation (Figure 7), and plasma cardiac enzyme levels to assess cell loss (Figure 8). In all three assays, 1 mg/kg iodide administration resulted in a significant improvement.

**Figure 9:**
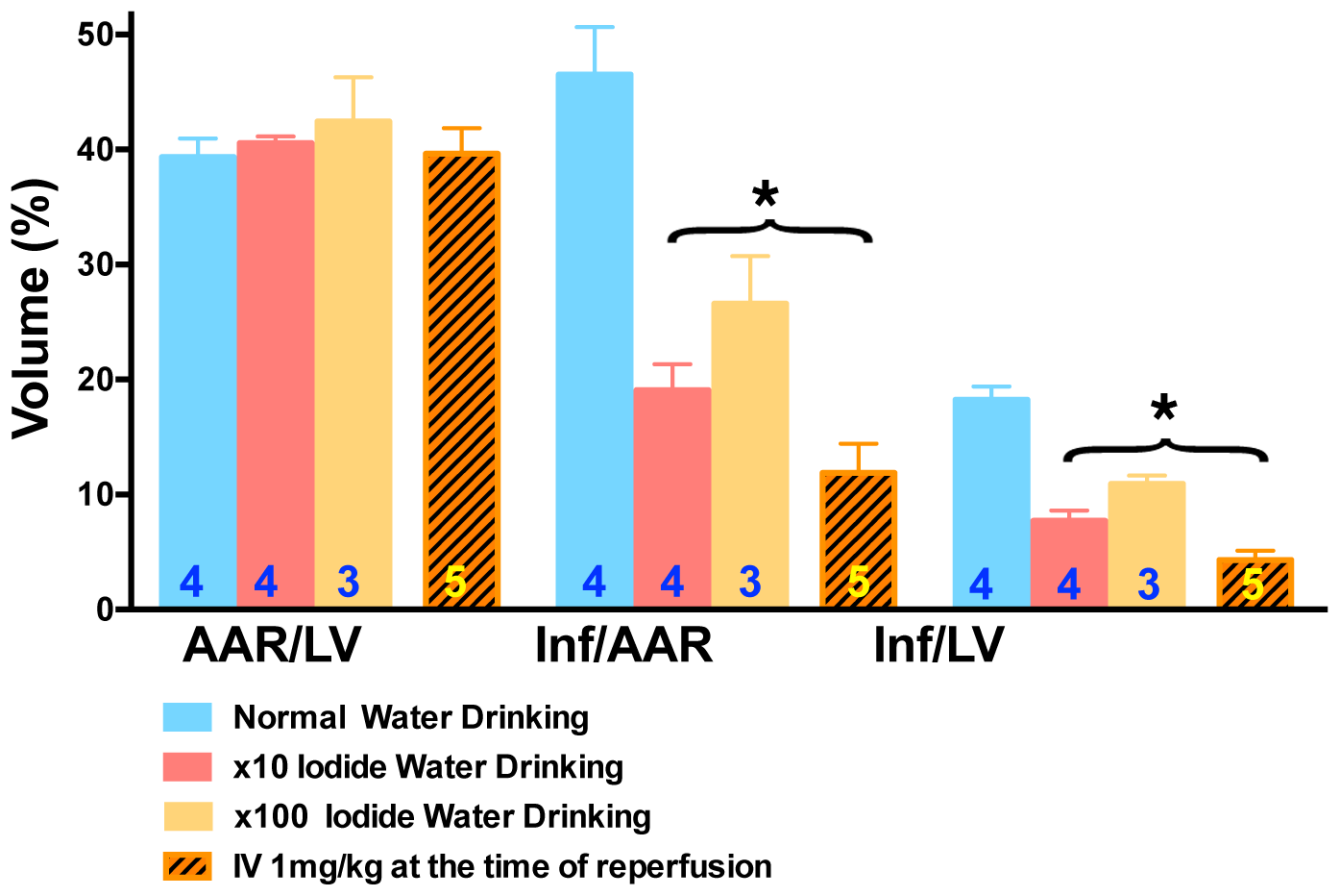
Orally administered iodide reduces heart damage after MIR. Infarct (Inf) and area at risk (AAR) volumes were measured using morphometry after administration of escalating doses of iodide. Y-axis is volume as a percent of total. X-axis labels are AAR/LV (ratio of area at risk volume to left ventricle volume), Inf/AAR (ratio of infarct volume to area at risk volume) and Inf/LV (infarct volume to left ventricular volume). The blue bars are hearts from normal water drinking animals. Red and tan bars are from animals that drank 0.028 mM or 0.28 mM iodide for 2 days prior to the MIR procedure, respectively. The orange hatched bars are from animals that received 1 mg/kg of iodide intravenously 5 minutes before reperfusion. Numbers in bars define group sizes. The two asterisk brackets over Inf/AAR and Inf/LV indicate that all 3 groups under the brackets show statistically significant improvements compared to control saline (p<0.05).

Previous research by other investigators shows that iodide is orally available. For this reason we tested whether iodide provided in drinking water could provide benefit in the MIR model. Animals were given iodide in their water for two days prior to the MIR procedure. The amount was such that the total iodine concentration in their diet was increased by either 10 or 100 times the amount in standard chow (normalized for consumption). At both concentrations we observed a significant decrease in heart damage as judged by morphometry (Figure 9).

**Figure 10:**
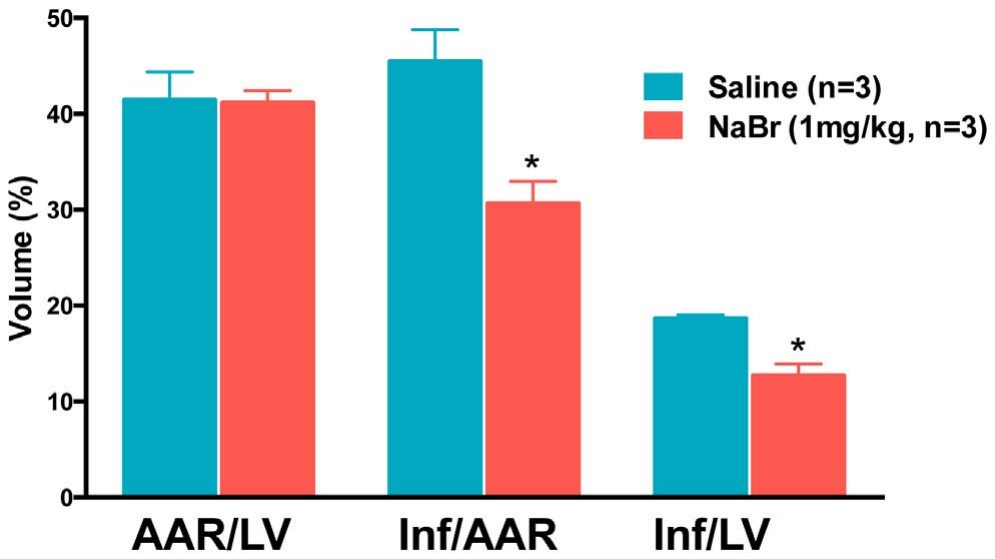
Bromide reduces damage after MIR. Infarct (Inf) and area at risk (AAR) volumes were measured using morphometry after administration of sodium bromide (1 mg/kg). Y-axis is volume as a percent of total. X-axis labels are AAR/LV (ratio of area at risk volume to left ventricle volume), Inf/AAR (ratio of infarct volume to area at risk volume) and Inf/LV (infarct volume to left ventricular volume Blue bars are saline and red bars are bromide. The asterisks indicate statistically significant differences between saline and bromide treated animals (p<0.05).

Because iodine is a halogen it was reasonable to test whether other halogens would behave similarly in the MIR assay: astatine, bromine and chlorine. Since astatine is not thought to be essential and only unstable isotopes exist, we did not test it in our assay. However, bromine was recently shown to be essential for life; it is required for the activity of peroxidasin in fruit flies [11]. Using sodium bromide (1 mg/kg, referred to here as bromide or NaBr) in our assay, we found that while there is a significant reduction in infarct size, the degree of reduction isless than that seen for iodide (Figure 10). We could not test chloride at the 1 mg/kg concentration we used for iodide and bromide as it was our control saline for these experiments, and in vivo concentration of chloride in blood exceeds 50 mg/kg.

To understand physiologic changes that may occur following iodide infusion we measured heart rate, body core temperature and carbon dioxide production. We found that a 1 mg/kg bolus intravenous iodide infusion resulted in a transient decrease in heart rate, oxygen consumption, and core body temperature when compared to saline infused control animals (Table 1).

**Table 1:** Iodide reduces metabolism.

	Pre			30 minutes after			150 minutes after		
	HR	CO_2_	Temp	HR	CO_2_	Temp	HR	CO_2_	Temp
**Saline**	**619 +/− 0.7**	**1121 +/− 277**	**36.7 +/− 0.5**	**643 +/− 95**	**2656 +/− 260**	**36.6 +/− 0.4**	**588 +/− 63**	**1750 +/− 494**	**36.2 +/− 0.4**
**Iodide**	**618 +/− 52**	**983 +/− 178**	**36.1 +/− 0.3**	**436 +/− 13**	**1745 +/− 165**	**34.0 +/− 0.9**	**597 +/− 78**	**1336 +/− 516**	**35.3 +/− 0.97**
**p value**	**0.5**	**0.07**	**0.015**	**0.03**	**0.004**	**0.0006**	**0.27**	**0.13**	**0.047**

Heart rate and body core temperature were measured using radiotelemetry. Carbon dioxide production was measured using an infrared CO_2_ analyzer. Heart rate values (HR) are expressed in beats per minute. CO_2_ values are ppm. Body core temperature values (Temp) are expressed in degrees Celsius. Measurements were made for 30 minutes before iodide or saline administration (pre) to determine baseline metabolism, at 30 minutes after iodide/saline administration and at 180 minutes after administration. Values shown are average +/− standard deviation and p value.

Because thyroid synthesis is the main iodine-dependent process in mammals, we tested whether decreasing thyroid function would affect the ability of iodide to reduce reperfusion injury. We fed animals an iodine deficient diet containing propylthiouracil; this diet is widely used to restrict thyroid function [12].

**Figure 11:**
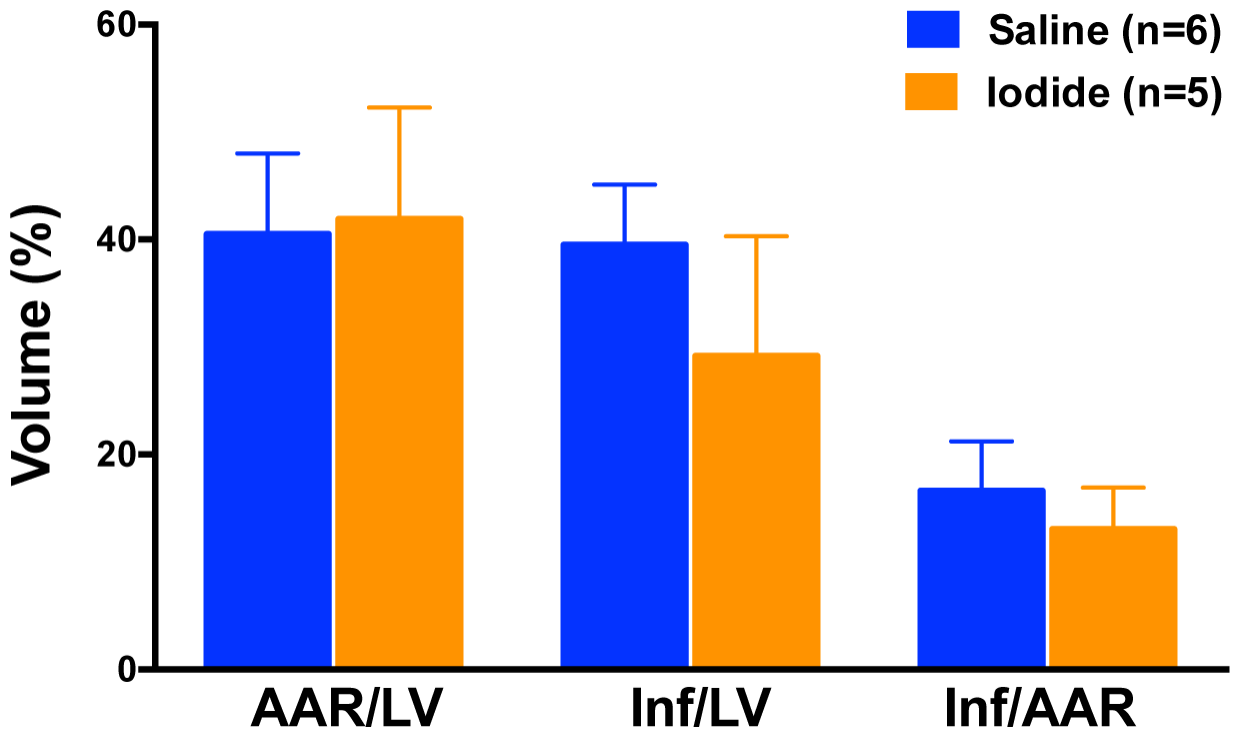
Thyroid function is required for iodide benefit. Mice fed iodide deficient chow containing 0.15% propylthiouracil were subjected to the MIR procedure with 2 hours of reperfusion. Infarct (Inf) and area at risk (AAR) volumes were measured using morphometry after administration of iodide. Y-axis is volume as a percent of total. X-axis labels are AAR/LV (ratio of area at risk volume to left ventricle volume), Inf/AAR (ratio of infarct volume to area at risk volume) and Inf/LV (infarct volume to left ventricular volume). Blue bars are values from animals administered saline; orange bars are values from iodide treated animals. The difference in infarct volume between saline and iodide treated animals is not significant.

We found that animals fed this diet for 3 months no longer benefitted from administration of iodide in the MIR model suggesting that normal thyroid function is required for iodide benefit (Figure 11).

## Discussion

Since its discovery in 1811, iodine has been intensively studied. Arguably the first human trial on a defined chemical showed in 1826 that iodine could be used to prevent and treat goiter [13]. Since then, a great deal of work has led to the understanding that iodine in the form of iodide is required for thyroid hormone synthesis [14]. The data presented here describe a new function for the iodide anion; intravenous infusion of iodide provides benefit from reperfusion injury suffered by heart tissue in a model of acute myocardial infarction.

As such, it is notable that iodide, but not iodate, provides this benefit. Both molecules are highly related and easily interconverted in the blood [15]. Iodate is not taken up by the thyroid but first enters red blood cells where it is reduced to iodide and then re-enters the plasma where it then is concentrated in the thyroid as well as several other tissues. One possibility for why we do not see these benefits with iodate is that during reperfusion, iodate conversion to iodide is impeded. There is evidence that the reducing environment of the red cell cytoplasm (required to reduce iodate to iodide) is compromised during acute myocardial infarction [16]. Further work will be required to confirm this possibility.

The involvement of normal thyroid function for iodide benefit suggests the possibility that thyroid hormone, thyroxine or one of its metabolites, might be affected by intravenous injection of iodide. Previous work shows that injection of the amounts of iodide beneficial in our MIR model are the same as those known to reduce thyroid hormone synthesis and secretion [17]. This effect, known as the Wolff-Chaikoff effect, has been observed in humans and animals [18]. Given that the hydrogen peroxide produced by thyroperoxidase (TPO) is required to oxidize iodide making it a substrate for thyroid hormone synthesis it has been suggested that excessive blood iodide may overwhelm the hydrogen peroxide produced by TPO thereby decreasing thyroid hormone production [19]. Because thyroid hormone is know to stimulate metabolism it is parsimonious to suggest that the benefit of iodide may be conferred by depressing thyroid hormone production and as a result decreasing cardiac metabolism that is linked to reperfusion injury. Further work will be required to test this hypothesis.

The fact that both iodide and bromide reduce reperfusion injury but that the effect is much greater with iodide is consistent with this model. Bromide is not well concentrated into the thyroid [20] and therefore less able to depress thyroid function than iodide. Furthermore, this result suggests that halogens may possess a graded activity. The trend toward decreasing electronegativity from smaller to larger halogens has been suggested as a reason why iodide was evolutionarily selected for inclusion in thyroid hormone [21].

Regardless of the mechanism of action, the utility, as defined by safety and efficacy of iodide, is hard to overstate. A comprehensive toxicology report written by the Agency for Toxicology Substances and Disease Registry in 2004 shows that it is possible to raise the concentration of iodide in humans from under 0.1 µM to higher than 1 mM in plasma without toxic effects [22]. Furthermore, studies show that iodide is more than 95% bioavailable when taken orally and that people can safely consume gram quantities every day for months – approximately 10,000 times the recommended daily allowance – without toxicity [23]. The ratio of the amount of iodide required for benefit compared to the toxic dose or therapeutic index (TI) shows that the TI for intravenous iodide in our MIR model in mice is over 100. Given this combination of safety and efficacy it is reasonable to further explore iodide as a treatment for acute myocardial infarction as well as other injuries and diseases involving reperfusion and excessive inflammation.
