# Correction: Iodide Protects Heart Tissue from Reperfusion Injury

**DOI:** 10.1371/journal.pone.0138396

**Published:** 2015-09-14

**Authors:** Akiko Iwata, Michael L. Morrison, Mark B. Roth

The authors wish to amend the Competing Interests statement for this article, which should have included additional information in relation to potential competing interests relevant to this work. The authors apologize for this omission and revise the Competing Interests statement to read as below.

The authors have read PLOS ONE's competing interests policy, and they have the following to declare: Dr. Roth is President, Founder, and a member of the Board of Directors for Faraday Pharmaceuticals, a company dedicated to promoting iodide therapy for ischemic injury. Dr. Roth and his co-authors, Drs. Iwata and Morrison, own stock in the company. Additionally, the authors are named on pending patent applications for iodide therapy (Use of Halogen Compounds For the Treatment and Prevention of Heart Attack and Ischemic Injury). This does not affect or alter our adherence to all of PLOS ONE's policies on sharing data and materials.
